# Zonation related function and ubiquitination regulation in human hepatocellular carcinoma cells in dynamic vs. static culture conditions

**DOI:** 10.1186/1471-2164-13-54

**Published:** 2012-02-01

**Authors:** Shu Cheng, Jean-Matthieu Prot, Eric Leclerc, Frédéric Y Bois

**Affiliations:** 1 Chaire de Modélisation Mathématique pour la Toxicologie Systémique, Université de Technologie de Compiègne, BP 20529, 60205 Compiègne Cedex, France; 2 CNRS UMR 6600, Laboratoire de Biomécanique et Bioingénierie, Université de Technologie de Compiègne, BP 20529, 60205 Compiègne Cedex, France; 3 INERIS, DRC/VIVA/METO, Parc ALATA, BP 2, 60550 Verneuil en Halatte, France

**Keywords:** hepatocellular carcinoma, hepatic zonation, ubiquitination system, gene sets analysis, Petri plates, PDMS microfluidic biochips

## Abstract

**Background:**

Understanding hepatic zonation is important both for liver physiology and pathology. There is currently no effective systemic chemotherapy for human hepatocellular carcinoma (HCC) and its pathogenesis is of special interest. Genomic and proteomic data of HCC cells in different culture models, coupled to pathway-based analysis, can help identify HCC-related gene and pathway dysfunctions.

**Results:**

We identified zonation-related expression profiles contributing to selective phenotypes of HCC, by integrating relevant experimental observations through gene set enrichment analysis (GSEA). Analysis was based on gene and protein expression data measured on a human HCC cell line (HepG2/C3A) in two culture conditions: dynamic microfluidic biochips and static Petri dishes. Metabolic activity (HCC-related cytochromes P450) and genetic information processing were dominant in the dynamic cultures, in contrast to kinase signaling and cancer-specific profiles in static cultures. That, together with analysis of the published literature, leads us to propose that biochips culture conditions induce a periportal-like hepatocyte phenotype while standard plates cultures are more representative of a perivenous-like phenotype. Both proteomic data and GSEA results further reveal distinct ubiquitin-mediated protein regulation in the two culture conditions.

**Conclusions:**

Pathways analysis, using gene and protein expression data from two cell culture models, confirmed specific human HCC phenotypes with regard to CYPs and kinases, and revealed a zonation-related pattern of expression. Ubiquitin-mediated regulation mechanism gives plausible explanations of our findings. Altogether, our results suggest that strategies aimed at inhibiting activated kinases and signaling pathways may lead to enhanced metabolism-mediated drug resistance of treated tumors. If that were the case, mitigating inhibition or targeting inactive forms of kinases would be an alternative.

## Background

The functional unit of the liver, the lobule, is roughly cylindrical, with branches of the hepatic artery and portal vein together with bile ducts at its periphery, and a central vein branch in the middle. Such a structure allows a natural distinction between upstream "periportal" and downstream "perivenous" hepatocytes, and constitutes the basis for "liver zonation". It is generally accepted that the homeostatic function of the liver depends on the functional complementation of those two zones [1-3]. Different, or even opposite, metabolic functions are often found between periportal and perivenous hepatocytes [4,5]. That heterogeneity is important for understanding of various aspects of liver function and pathology [2], and is widely studied [5-7]. However, little attention has been paid so far to zonation-like alterations associated with human hepatocellular carcinoma (HCC), which is one of the most frequent visceral neoplasms worldwide [8].

Accumulating evidence indicates that WNT/β-catenin signaling plays a central role in the multi-level regulation of zonal gene expression in the liver [5]. It is also implicated in several critical pathways responsible for initiating and maintaining dysregulated cell proliferation in HCC [9]. A key factor in the WNT/β-catenin signal transduction pathway is the success or failure of proteasomal degradation of the cytosol β-catenin. Migration of accumulated β-catenin to the nucleus subsequently induces the synthesis of several tumor promoters. In normal physiological conditions, β-catenin activation is restricted to perivenous hepatocytes [10,11], where it both induces "perivenous" gene expression and inhibits "periportal" gene expression [10]. Genetic therapy of HCC faces severe challenges, since HCC involves genetic alterations of multiple genes in several regulatory pathways simultaneously [8]. Understanding the relationships between the zonation of these pathways in the liver may deepen our views on the pathogenesis of HCC, its proliferation or maintenance.

The ubiquitin-proteasome system, a highly conserved eukaryotic system for cellular protein degradation, is important for cancer cells to block apoptosis or other normal cellular processes [9]. The levels of proteins in the nucleus, cytoplasm, endoplasmic reticulum lumen, and in membranes, are all kept in check by the ubiquitination enzymes and the proteasome [12]. A variety of healthy or diseased cell functions are affected by this regulatory mechanism. The corresponding signaling cascade includes the ubiquitin-activating enzyme E1, ubiquitin-conjugating enzymes E2 and a highly diverse class of ubiquitin ligases E3. The wide variety of E3 ligases, mainly including the RING-, SCF-, HECT- and U-box-types, target a tremendous number of proteins for ubiquitination. For example, c-Cbl E3 ligase ubiquitinates several receptor protein-tyrosine kinases [13,14]. Alterations in protein ubiquitination, such as modified affinity of a signaling receptor for E3 enzymes after phosphorylation of its intracellular domain, lead to dysregulated signaling, promotion of cancer cells' growth and their withstanding of anti-proliferative and cell death stimuli [14,15].

With the increasing availability of transcriptomic and proteomic data, pathway analysis has become a significant avenue to uncover the structure of functional/regulatory networks in tumoral or normal tissues. Pathway-based analyses are usually based on an assessment of correlations in genes or proteins expressions. Various algorithms, such as gene set enrichment analysis (GSEA) [16,17] and structural information based pathway analysis [18], have been developed to identify biologically relevant pathways. Those methods may take advantage of information previously gathered in databases such as KEGG (Kyoto Encyclopedia of Genes and Genomes, http://www.genome.jp/kegg/) or BioCarta to improve statistical power. A pathway approach has been able, for example, to identify a coordinated decrease in the expression of functional signals in human diabetes [16].

Meanwhile, technological advances in liver tissue engineering and biomechanics allow *in vitro *studies with much tighter control of extraneous conditions. Numerous *in vitro *hepatocyte models have been proposed [19]. Hepatocyte cultures are one of the most successful *in vitro *systems, since liver-specific functions and response to stressors can be maintained with appropriate culture conditions [7]. The major problems can be limited viability, associated with the loss of phase I and phase II biotransformation capacity [20]. "Biochips" grow cells in a microfluidic network, allowing a better control of dynamic cultures by continuous feeding of nutrients to cells and waste removal [21,22]. Previous transcriptomic analyses of hepatocyte function in dynamic biochips showed that phase I and phase II enzymes were significantly up-regulated and xenobiotic metabolism maintained [23].

In this study, we carried out an extensive GSEA pathway analysis of gene and protein expression data from HCC cells grown in either microfluidic biochips (μFB) or Petri dishes (PD), representing dynamic and static micro-environments, respectively. On the basis of these analyses, we investigated the relationship between activated pathways, experimental RT-qPCR data, and daily hepatocytes' metabolic activities [23], in the framework of hepatic zonation. Finally, the distinct effects of matched protein expression data in the two groups led us to investigate the role of ubiquitination in the onset of oncogenic properties.

## Results

### Different categories of pathways are activated in μFB and PD

We performed GSEA [16,17] of gene transcription and protein expression data obtained in human HCC cell lines cultured in μFB and PD. Gene expression data were used alone or in combination with matched protein expression data. The pathways definitions used were those of the KEGG database. Table 1 categorizes the gene sets differentially expressed in the μFB and PD groups with a false discovery rate (FDR) ≤ 0.25 (they mostly have a nominal *p *value < 0.05). The number of pathways found and their specificities were quite different between the two culture conditions. A pronounced metabolism-pathways profile was found in μFB, while a signaling-pathways profile (referring to environmental information processing) dominated in PD (see Additional file 1, Table S1 and Additional file 2, Table S2 for a detailed list of the pathways). That result is supported by the observation that, compared to other KEGG pathway categories, signaling and regular metabolic pathways have a general tendency to lose their gene expression coherence in tumor cells [24]. Typical liver-function-specific pathways (e.g., metabolism of xenobiotics by cytochrome P450 and primary bile acid biosynthesis) and cancer-related pathways (e.g., MAPK signaling) were significantly activated in the μFB and PD groups, respectively. The result on metabolic pathways in μFB is supported by [23], and indicates that μFB cultures represent a 3D culture condition yielding metabolically competent cells, closer in phenotype to primary human hepatocytes [22,25]. Among the metabolic pathways activated in μFB, only one out of 29 concerned energy metabolism (i.e., oxidative phosphorylation), which has been shown to be mostly periportal [26] (Additional file 1, Table S1). Only nitrogen energy metabolism was activated in PD (Additional file 2, Table S2). Genetic information processing pathways, which are highly correlated with genomic alterations in HCC [8], were the second most activated in μFB while the corresponding information was lost in PD (Table 1). Previous experiments in mice liver lobules suggest that the perivenous genetic program is switched off, and the periportal program switched on, by a WNT inhibitor or by lowered expression of β-catenin [26]. Using the same gene expression data, there were 11 pathways with an FDR ≈ 0 in μFB (data not shown) and only one in PD: "maturity onset of diabetes of the young", a monogenic form of type 2 diabetes [27], which is in agreement with our cell model.

**Table 1:** Statistical summary by categories of the KEGG gene pathways identified by GSEA (FDR ≤ 0.25), on the basis of differential gene and protein expressions between μFB and PD cultured HepG2/C3A cells

Pathway category	μFB cultures	PD cultures
Metabolism	29/25 ***^a^***	1/2

Genetic Information Processing	8/7	0/0

Environmental Information Processing	1/1	6/7

Cellular Processes	3/3	1/1

Organismal Systems	2/2	5/5

Human Diseases	1/2	4/7

Total	44/40	17/22

*^a ^*results using gene expression data only/results using gene *and *protein expression data.

Results with and without protein data included were not very different, due to the much lower number of identified relevant proteins. However, close examination showed that the effects of protein data inclusion were quite opposite in the μFB and PD groups: In μFB, the total number of pathways was reduced, although new pathways such as RIG-I-like receptor signaling were activated; In PD, an increased number of pathways were identified (Additional file 1, Table S1 and Additional file 2, Table S2).

### HCC-related CYPs and kinases represent the cancer phenotypes in μFB and PD, respectively

We proceeded to assess whether one of the above identified pathways offered a clear separation of the HCC phenotypes in μFB and PD. That is especially useful for manually curated gene sets like KEGG pathways, which may represent amalgamated processes [17]. GSEA was used to examine, at the gene level, the leading-edge pathways identified above. Table 2 presents the top genes participating to at least four pathways identified using both gene and protein expression data (the detail of the genes and connected pathways in μFB using gene expression data are shown in Additional file 3, Figure S1). Eleven and 16 genes are included in the μFB and PD, respectively. Overall, there is notably more gene sharing in the top 22 PD pathways than in the top 40 μFB pathways, indicating a high density of pathway cross talk in PD, consistent with the least cohesive property of signaling pathways [24]. Yet, in both groups, many genes belong to a common gene family. In μFB cultures, four (one CYP1A and three CYP3A) out of 11 genes are the members of cytochrome P450 superfamily. HCC has effects on the expression of CYP1A and CYP3A genes [28,29]. In particular, CYP3A4 shows a notable increase in gene copies and mRNA transcripts in HCC cell lines from eight ethnically diverse human populations [30]. In PD cultures, twelve of the genes found (BRAF, MAPK3, MYC, TGFB2, EGFR, CDC42, PDGFRA, CREBBP, PDGFB, RAP1A, PAK1, PRKCG), out of 16, are manifested in hepato-carcinogenesis [9]. They belong to kinases and proteins having kinase activities, growth factors and transcription factors playing critical roles in HCC, and several of them are targets of drug currently tried for HCC treatment.

**Table 2:** Genes ***^a ^***present in at least four of the pathways analyzed in Table 1, using gene and protein expression data together

			Genes					Number of
	μFB cultures				PD cultures			pathways
**CYP3A43**	**CYP3A4**	**CYP3A7**						6
POLD3	POLD2							

ALDH3A2	ALDH2				**BRAF**			5

POLE2	POLE3	RPA2	**MAPK3**	MYC	PPP3CC	IL5	TGFB2	
**CYP1A1**			**EGFR**	CACNA1C	CDC42	**PDGFRA**	CREBBP	4
			PDGFB	RAP1A	**PAK1**	**PRKCG**	PPP3RR2	

***^a ^***Genes in bold font code for HCC-related CYPs in the μFB group, and for kinases (or protein having kinase activity) in the PD group.

### Periportal- and perivenous-like pathways characterize μFB and PD, respectively

Although specific liver functions and HCC signals are more or less expressed in the two groups, the overall μFB and PD cellular phenotypes are quite different. Previous studies indicate that the loss or gain of β-catenin signaling has important consequences; In the former case, liver cells acquire a periportal-like phenotype [26]. We thus postulated that the pathways patterns observed in the μFB and PD groups could be interpreted as the result of loss or over-activation of the β-catenin pathway, respectively. That was hinted at by the elevated expression levels of periportal-like and perivenous-like markers in the above GSEA results, but also in previously published RT-qPCR and metabolic activity data [23].

Under normal conditions, the expression of cytochromes P450 is mostly restricted to perivenous hepatocytes and under β-catenin regulation. The hypothesis that β-catenin signaling is localized in the perivenous area has been well described [5,10]. Evidence has been recently provided that, in β-catenin knockout mice, CYP1A and CYP3A expression is strongly altered [5,31]: High CYP3A mRNA and protein levels are observed in periportal hepatocytes, while CYP1A induction by AhR agonists occurred uniformly in all hepatocytes. In rat liver, low doses of β-naphtoflavone lead to CYP1A1 induction in periportal hepatocytes [32,33]. On the other hand, CYP3A zonation is completely lost in human HCC [34].

In the above GSEA analyses, only the CYP1A and 3A families (among the 60 CYP isoforms) were found in the top-ranking pathways for μFB cultures (Table 2 and Additional file 3, Figure S1). Similarly, RT-qPCR has shown up-regulation of CYP1A1, 1A2, 3A4, 3A5 and 3A7, with a slight induction of AhR transcription (for CYP1A) and PXR transcription (for CYP3A) in μFB cultures, compared to PD cultures [23].

**Figure 1:**
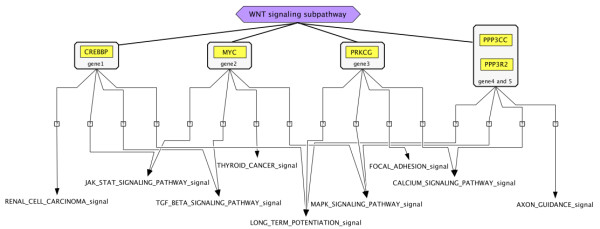
**A joint subset of five genes, each of which participates in the overlapping four pathways in the PD group, constitutes the WNT signaling sub-pathway (CREBBP, MYC, PRKCG, PPP3CC and PPP3R2)**. These genes might constitute the specific induction of WNT signaling in HepG2/C3A cells (originating from an adolescent's liver).

To visualize β-catenin signaling expression in PD cultures, the KEGG WNT signaling pathway was drawn by Paintomics [35] on the basis of the PD gene expression data (Additional file 4, Figure S2). Over-expressed genes dominate the painted WNT signaling pathway, with dense connections between WNT signaling and the pathways governing focal adhesion, MAPK, TGF-β and calcium signaling pathways, all activated in PD cultures. Other relevant expressions in PD cultures are the nitrogen metabolism pathway, controlled by β-catenin [10], and the EGFR gene, which enhances β-catenin transcriptional activity [36]. Furthermore, five genes, each of which participates in the overlapping four pathways in PD cultures, are involved in the WNT signaling pathway (CREBBP, MYC, PRKCG, PPP3CC and PPP3R2) (Figure 1). These genes might constitute the specific induction of WNT signaling in HepG2/C3A cells (originating from an adolescent's liver) although MYC is normally not induced by β-catenin signaling in the adult liver [26]. The absence of CYPs in HepG2/C3A PD cultures is consistent with the overall repressive effect of Ras-Raf-MAPK signaling on expression of CYP enzymes [37].

**Figure 2:**
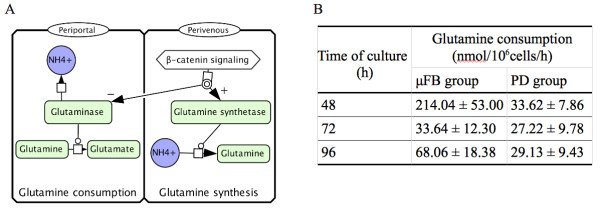
**Zonated glutamine cycling in the liver**. (A) β-catenin exerts either negative or positive control on glutaminase and glutamine synthetase, respectively. Periportal glutamine consumption is catalyzed by glutaminase. Perivenous glutamine synthesis is catalyzed by glutamine synthetase. (B) Daily glutamine consumption is higher in μFB cultures than in PD cultures. Data are expressed as mean ± SD (N = 6).

Differences in glutamine-related ammonia detoxification between μFB and PD cultures also maps to the spatial heterogeneity of hepatocytes *in vivo*. Periportal glutaminase and perivenous glutamine synthetase genes are respectively negatively and positively controlled by β-catenin [26] (Figure 2A). Therefore, the net glutamine balance across the liver, involving periportal consumption and perivenous glutamine synthesis, can be either positive, negative or zero, depending on experimental conditions [38]. Glutamine consumption was found to be higher in μFB than in PD cultures, especially after 48 h [23] (Figure 2B). Dynamic flow conditions in microfluidic bioreactors lead to increased glutamine consumption and ammonia production, compared to static bioreactor conditions or Petri dishes [39]. Glutaminase activity might therefore be more active in μFB as it is in the periportal area, while glutamine synthetase activity should dominate in PD, like in perivenous hepatocytes.

**Figure 3:**
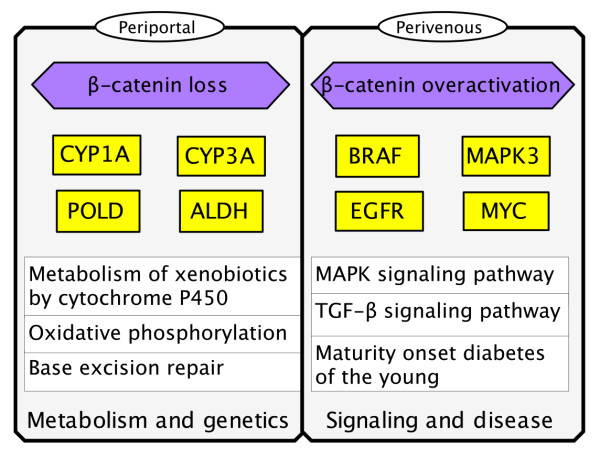
**Schematic representation of the activated signals corresponding to periportal-like HCC (μFB cultures) and perivenous-like HCC (PD cultures) as the result of loss or over-activation of β-catenin, respectively**. Genetic pathways are significantly induced under loss of β-catenin, leading to periportal-like phenotype (with production of energy by oxidative phosphorylation) in μFB hepatocytes. β-catenin-dependent signals are significantly enriched in the perivenous-like hepatocytes of PD cultures.

Taken together, those results suggest that the periportal- and perivenous-like pathways are differentially activated in dynamic μFB and static PD, respectively (Figure 3).

### Distinct ubiquitin-mediated regulations in μFB and PD cultured cancer cells

As mentioned above, including protein expression data in the initial GSEA had distinct effects on pathway inference for μFB and PD cultures (Table 1, Additional file 1, Table S1 and Additional file 2, Table S2). That suggests that the coordination of gene and protein expressions is somewhat different in the two culture types. Post-transcriptional mechanisms, such as protein degradation, are among the possible explanations for that observation. We thus explored a possible role of ubiquitination in leading to the observed cell phenotypes (Additional file 5, Table S3) and GSEA results.

**Figure 4:**
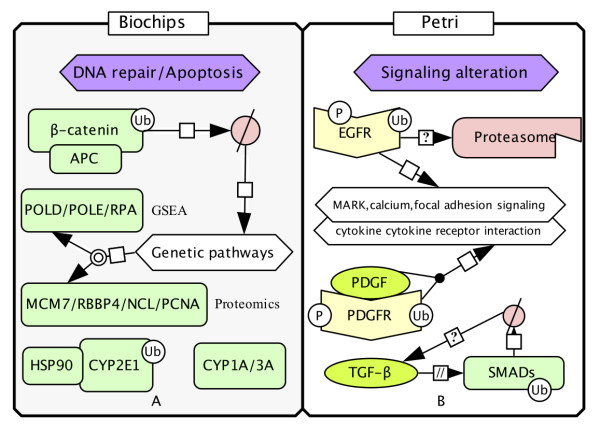
**Distinct ubiquitination-mediated proteins regulation in the μFB (Biochips) and PD (Petri) cultured HCC cells**. (A) regulation through genetic alteration coupling with proteasomal degradation and apoptosis. (B) regulation through signaling alteration by signaling proteins ubiquitination.

In μFB cultures, there appears to be a loss of β-catenin pathway activity, which might correlate to the activated "proteasomal" (Additional file 1, Table S1) digestion of β-catenin. That degradation has been shown to be dependent on the shuttling of APC [40] (Figure 4). The loss of β-catenin regulation might favor the activation of genetic pathways over signaling pathways. Almost 50% of the top genes differentially expressed in μFB cultures (POLD, POLE and RPA genes) (Table 2) are involved in chromosomal DNA replication. Proteomic analysis showed an elevated protein expression of DNA repair (including MCM7, RBBP4, NCL and PCNA) in μFB cultures. In addition, increasing evidence links proteasome function with chaperones [12]. An observed over-expression of HSP90AA1 and AB1 proteins might be in response to the degradation of CYP2E1 [41]. GSEA excluded CYP2E1 from the list of enriched genes in the "metabolism of xenobiotics by cytochrome P450" and "drug metabolism cytochrome P450" pathways (data not shown). That result is in accordance with the HCC database [42] which shows that almost half of the specimens of CYP2E1 are either up- or down-regulated in a ratio of 10 to 339. Overall, in μFB cultured HCC cells, a high degree of genetic instability seems to facilitate the inactivation of normal and tumor suppressor proteins as well as the activation of HCC-related proteins like CYP1A and CYP3A.

In PD cultures, E3 ubiquitin ligases RNF2 and RNF20, cathepsins B, C and D, all of which are involved in the ubiquitination system, had significantly elevated expression (Additional file 5, Table S3). They may play a major role in directing the signaling proteins singled out by GSEA to ubiquitination and lysosomal degradation (Figure 4 and Table 2). For instance, it has been clearly shown that the RING finger ligase can be recruited by EGFR for subsequent routing to lysosomal degradation [43]. Yet, in most cases, ubiquitinated EGFR activates the internalization and is not targeted by the proteasome [12]. The altered proteolytic machinery may imply an ubiquitin modulation of EGFR, which could be associated with distinct stages in the transition to the active kinase forms of EGFR [44,45]. That may serve as a temporal/spatial control of EGF signaling, which finely regulates pathways related to survival, proliferation and angiogenesis. Similarly, the receptor tyrosine kinase PDGFRA, together with its ligand, participates to these pathways and is a substrate of c-Cbl E3 ubiquitin ligase [14,46]. The TGF-β ligand itself plays an important role in cancer progression by functioning both as an anti-proliferative factor and as a tumor promoter [47], and the ubiquitin-proteasome system is known to regulate the core intracellular signaling cascade SMAD proteins [9,12,47]. Therefore, in contrast to the μFB culture phenotype which may result from genetic alterations, the PD cultured HCC cell phenotype could be influenced by an altered ubiquitination of signaling proteins.

## Discussion

The emerging consensus on hepatic zonation is that it spatially separates pathways to avoid interference and energy wastage [48]. This notion is compatible with our proposed parallel between zonation profiles and culture types: Upstream hepatocytes, as in biochips cultures, exhibit broad metabolic and genetic profiles; Downstream hepatocytes, as in plates, display mainly signaling and disease processes.

Previous studies have pointed to several limitations of HepG2 hepatocytes: A repression of various CYP isoforms by the EGF/Ras/MAPK signaling transduction pathway [37]; The development of a periportal genetic program, allowed by inhibition of WNT signaling [10]; The absence of a urea cycle, leading to ammonia detoxification only *via *glutamine synthesis [49]. The first two limitations are clearly culture-type dependent, as shown by our analysis of μFB and PD culture conditions. As for the third limitation, our study indicates that favoring a periportal-like phenotype in μFB cultured hepatocytes might improve urea synthesis in HepG2 cells. We assert that the causes of these limitations are linked to the β-catenin pathway. That, however, remains to be experimentally confirmed, because other regulatory pathways might be involved as well. It would be especially interesting, for example, to study wild-type CTNNB1 hepatoma cells which express β-catenin and metabolic enzymes at levels even closer to the *in vivo *situation.

It is interesting to note that HCC-related CYPs were activated in μFB cultures, where a deficit in signaling pathways was observed. On the other hand, protein kinases were activated in PD cultures, with a serious deficit in metabolic pathways. It seems reasonable to assume that both pathway categories need to be balanced, and that imbalance leads to homeostasis abnormalities. That is important because much efforts are devoted to developing kinases and signaling pathways inhibitors for therapeutic intervention. The results presented here indicate that in such conditions, cancer cells may spontaneously develop metabolism-mediated resistance. In that case, it might be worth to mitigate inhibition of the active form of kinases, or to target inactive kinase conformations (*e.g*., PDGFR and BRAF inhibitors [50]). The initial stage of inactivation of a typical cancer-related kinase (PKB/AKT) has been recently described [45].

Protein phosphorylation and ubiquitination go hand in hand in the regulation of many cellular processes, and phosphorylation typically precedes ubiquitination [12]. It is commonly thought that the loss of apoptosis is required for carcinogenesis, and that cancer cells aim to develop survival and growth. However, different environmental changes, which can be related to personalized medicine, can significantly modulate the cells responses such as the genetic alteration coupled with proteasomal degradation and apoptosis in μFB cultured HCC cells, and signaling alteration in PD cultures. Moreover, the fact that ubiquitinated EGFR is in most cases not targeted to the proteasome may indicate that signaling pathways remain functional despite the ubiquitination of their proteins, but this remains to be investigated.

## Conclusions

This paper describes links between β-catenin controlled hepatic zonation and the phenotype of HepG2 cells grown in dynamic μFB cultures or static PD conditions, on the basis of gene and protein expression data. CYP1A/3A and kinases are representative of the cancer phenotypes in μFB and PD, respectively. Plausible ubiquitin-mediated regulation mechanisms are proposed for β-catenin degradation in μFB, and ubiquitin alteration of signaling proteins in PD.

## Methods

### Hepatocyte cell line

The human HCC cell line HepG2/C3A used was from the American Type Culture Collection (ATCC), number CRL-10741. HepG2/C3A was derived from the liver tissue of a fifteen year old male. C3A is a clonal derivative of HepG2 that was selected for strong contact inhibition of growth, high albumin production, high production of alpha fetoprotein (AFP) and ability to grow in glucose deficient medium http://www.atcc.org.

### Cell culture conditions: biochips and Petri

The microfluidic biochip fabrication, based on polydimethylsiloxane (PDMS), is detailed in [23,39].

The biochips had a volume estimated at 40 μL and the total surface available to cell growth was about 2 cm^2^. The inner surface of the biochips was coated with 10 μg/mL fibronectin for 40 min. The cells were inoculated in the biochip at the density of 0.25·10^6 ^cells/cm^2 ^in 0.5 ml medium and then kept at rest 24 h in a 5% CO2 incubator at 37°C. The culture medium was perfused at a 10 uL/min flow for 72 h and altogether 96 h for cells in the cultures.

In Petri cultures, the cells were seeded at the same density as in biochips, in 12-well culture plates, containing 2 mL medium covered initially with 0.5 mL PDMS and coated with fibronectin as in the biochips. Cells were cultivated for a total of 96 h including the first 24 h of adhesion phase.

### Transcriptional and protein level datasets

The gene expression data were generated using Affymetrix Genechip microarrays. The relevant technique is detailed in [23]. The GEO access of the data is GSE27420. The protein level data were obtained via 2D DIGE technique and are detailed in Additional file 5, Table S3.

There were 21380 differentially expressed genes in each of the triplicates biochips and Petri experiments. The average coefficient of variation (CV) of the triplicate gene expression measurements was 2.6% in biochips and 2.4% in Petri plates; The 95th percentile of the CVs of all genes was 6.6% in biochips and 6.3% in Petri. The variability across replicates was therefore fairly low and for the subsequent analyses we used the mean expression values of the triplicates for each gene. Only 101 proteins were identified. The ratio of biochip to Petri expression was used for biochips and the value "1" assigned to all the Petri protein expressions.

### Gene set enrichment analysis (GSEA)

GSEA determines whether the members of a given gene set are enriched among the most differentially expressed genes between two classes. Calculation of the enrichment score *ES *is the key component of GSEA, which is given by

(1) $$P_{hit}\left(S,i\right)=\underset{g_{j}\in S,j\leq i}{\sum }\frac{{\left(r_{j}\right)}^{p}}{N_{R}},\text{where}\;N_{R}=\underset{g_{j}\in S}{\sum }{\left(r_{j}\right)}^{p}$$

(2) $$P_{miss}\left(S,i\right)=\underset{g_{j}\notin S,j\leq i}{\sum }\frac{\text{1}}{N-N_{H}}$$

where a gene set *S *has *N_H _*genes which forms the ranked order *L *= (*g*_1_, ..., *g_N_*) according to the correlation, *r*(*g_i_*) = *r_j _*of their expression profiles with a phenotype C, and the *ES *is the maximum deviation from zero of *P_hit _- P_miss_*, representing the fraction of genes in *S *weighted by their correlation minus the fraction of genes not in *S*. The false discovery rate (FDR) was used for significance testing. GSEA is implemented as a software tool; for details see [16,17]. We ran GSEA using the unfiltered data, as recommended.

*WNT signaling pathway visualization*. Paintomics [35] for KEGG pathway visualization was used to display the WNT signaling pathway; Pathway enrichment measure was based on the Fisher's exact test. The gene expression data from PD were first log-transformed and normalized by subtraction of the mean value of all genes. Genes with values higher than the mean value were colored in red and in blue otherwise, while the intensity was proportional to the differentially expressed level.

## Conflicts of interests

The authors declare that they have no competing interests.

## Authors' contributions

SC drafted the paper, did GSEA and mechanisms investigation. JMP did the experiments and data analysis, and made revisions. EL is the project leader of the microfluidic biochip research and was involved in the experimental setting up, data analysis and made revisions. FYB supervised the data analysis and writing of the paper, fully interacting with the other authors. All authors have read and approved the final manuscript.

## Supplementary Material

Additional file 1 **Table S1: Specific pathways and involved gene counts in the biochips group using two expression data sets (FDR ≤ 0.25)**. The results are from GSEA using KEGG database. Click here for file

Additional file 2 **Table S2: Specific pathways and involved gene counts in the Petri group using two expression data sets (FDR ≤ 0.25)**. The results are from GSEA using KEGG database. Click here for file

Additional file 3 **Figure S1: The detailed top common genes of POLD(A), CYP3A(B), ALDH and EHHADH(C) together with their connected pathways in the biochips group using gene expression data**. Click here for file

Additional file 4 **Figure S2: Painted WNT signaling pathway based on the Petri gene expression data using Paintomics**. Genes with values higher than the mean value of all gene expressions are colored in red and otherwise in blue, while the intensity is proportional to the differentially expressed level. The image shows the link-outs to other pathways as well. Click here for file

Additional file 5 **Table S3: Proteins differentially expressed by the biochips versus the Petri via 2D DIGE technique**. Click here for file
