# Supplementary material for: Zonation related function and ubiquitination regulation in human hepatocellular carcinoma cells in dynamic vs. static culture conditions
Source: BMC Genomics. 2012 Feb 1;13:54. doi: 10.1186/1471-2164-13-54 (PMC3295679; doi:10.1186/1471-2164-13-54)
Supplement: Additional file 1 — Table S1: Specific pathways and involved gene counts in the biochips group using two expression data sets (FDR ≤ 0.25). The results are from GSEA using KEGG database. [file 1471-2164-13-54-S1.PDF]

Table 1 Specific pathways and involved gene counts in the biochips group using two expression data sets (FDR≤0.25). The results are from GSEA using KEGG database.

**Pathways**	# members	# members in signal (gene)	# members in signal (gene+protein)
ABC_TRANSPORTERS	42	10	10
AMINOACYL_TRNA_BIOSYNTHESIS	36	25	25
APOPTOSIS	83	27	27
BASE_EXCISION_REPAIR	32	15	14
BETA_ALANINE_METABOLISM	22	10	9
BIOSYNTHESIS_OF_UNSATURATED_FATTY_ACIDS	22	13	13
BUTANOATE_METABOLISM	30	8	5
CELL_CYCLE	115	46	46
CITRATE_CYCLE_TCA_CYCLE	29	10	
DNA_REPLICATION	35	19	18
DRUG_METABOLISM_CYTOCHROME_P450	51	15	14
DRUG_METABOLISM_OTHER_ENZYMES	33	11	11
FATTY_ACID_METABOLISM	38	18	
GLUTATHIONE_METABOLISM	46	21	21
GLYCEROLIPID_METABOLISM	45	19	18
GLYCINE_SERINE_AND_THREONINE_METABOLISM	29	11	7
GLYCOSYLPHOSPHATIDYLINOSITOL_GPI_ANCHOR_BIOSYNTHESIS	23	7	7
HOMOLOGOUS_RECOMBINATION	26	13	13
LINOLEIC_ACID_METABOLISM	24	5	5
METABOLISM_OF_XENOBIOTICS_BY_CYTOCHROME_P450	52	10	10
MISMATCH_REPAIR	21	11	13
N_GLYCAN_BIOSYNTHESIS	41	24	24
NUCLEOTIDE_EXCISION_REPAIR	39	17	16
ONE_CARBON_POOL_BY_FOLATE	16	3	3
OXIDATIVE_PHOSPHORYLATION	106	36	
PEROXISOME	70	27	27
PHENYLALANINE_METABOLISM	16	4	
PORPHYRIN_AND_CHLOROPHYLL_METABOLISM	29	16	16
PRIMARY_BILE_ACID_BIOSYNTHESIS	16	5	5
PROPANOATE_METABOLISM	31	12	10
PROTEASOME	43	34	34
PROTEIN_EXPORT	22	8	
PROXIMAL_TUBULE_BICARBONATE_RECLAMATION	21	3	
PYRIMIDINE_METABOLISM	87	33	32
PYRUVATE_METABOLISM	38	16	13
RETINOL_METABOLISM	44	6	5

RIG_I_LIKE_RECEPTOR_SIGNALING_PATHWAY	63		22
SELENOAMINO_ACID_METABOLISM	23	5	5
SPHINGOLIPID_METABOLISM	37	8	8
STEROID_BIOSYNTHESIS	16	12	12
STEROID_HORMONE_BIOSYNTHESIS	41	8	8
SYSTEMIC_LUPUS_ERYTHEMATOSUS	108	31	31
TOLL_LIKE_RECEPTOR_SIGNALING_PATHWAY	89	29	29
TRYPTOPHAN_METABOLISM	37	11	9
VALINE_LEUCINE_AND_ISOLEUCINE_DEGRADATION	41	19	24
VIBRIO_CHOLERAE_INFECTION	48		21
