# Supplementary material for: Zonation related function and ubiquitination regulation in human hepatocellular carcinoma cells in dynamic vs. static culture conditions
Source: BMC Genomics. 2012 Feb 1;13:54. doi: 10.1186/1471-2164-13-54 (PMC3295679; doi:10.1186/1471-2164-13-54)
Supplement: Additional file 2 — Table S2: Specific pathways and involved gene counts in the Petri group using two expression data sets (FDR ≤ 0.25). The results are from GSEA using KEGG database. [file 1471-2164-13-54-S2.PDF]

Table 2 Specific pathways and involved gene counts in the Petri group using two expression data sets (FDR≤0.25). The results are from GSEA using KEGG database.

**Pathways**	# members	# members in signal (gene)	# members in signal (gene+protein)
ASTHMA	24	14	14
AXON_GUIDANCE	124	47	47
CALCIUM_SIGNALING_PATHWAY	170	60	60
CELL_ADHESION_MOLECULES_CAMS	115	25	25
COMPLEMENT_AND_COAGULATION_CASCADES	65	25	19
CYTOKINE_CYTOKINE_RECEPTOR_INTERACTION	239	93	93
FOCAL_ADHESION	185	61	61
HEMATOPOIETIC_CELL_LINEAGE	80	21	21
HYPERTROPHIC_CARDIOMYOPATHY_HCM	80		40
JAK_STAT_SIGNALING_PATHWAY	142		36
LONG_TERM_POTENTIATION	66	14	14
MAPK_SIGNALING_PATHWAY	250	103	103
MATURITY_ONSET_DIABETES_OF_THE_YOUNG	24	11	11
NEUROACTIVE_LIGAND_RECEPTOR_INTERACTION	259	109	109
NITROGEN_METABOLISM	21	5	5
OLFACTORY_TRANSDUCTION	348	153	153
PATHOGENIC_ESCHERICHIA_COLI_INFECTION	45	10	10
RENAL_CELL_CARCINOMA	65		17
STARCH_AND_SUCROSE_METABOLISM	34		15
TGF_BETA_SIGNALING_PATHWAY	82	39	39
THYROID_CANCER	27	5	5
TYPE_I_DIABETES_MELLITUS	31		7
