# Supplementary material for: Zonation related function and ubiquitination regulation in human hepatocellular carcinoma cells in dynamic vs. static culture conditions
Source: BMC Genomics. 2012 Feb 1;13:54. doi: 10.1186/1471-2164-13-54 (PMC3295679; doi:10.1186/1471-2164-13-54)
Supplement: Additional file 3 — Figure S1: The detailed top common genes of POLD(A), CYP3A(B), ALDH and EHHADH(C) together with their connected pathways in the biochips group using gene expression data. [file 1471-2164-13-54-S3.PDF]

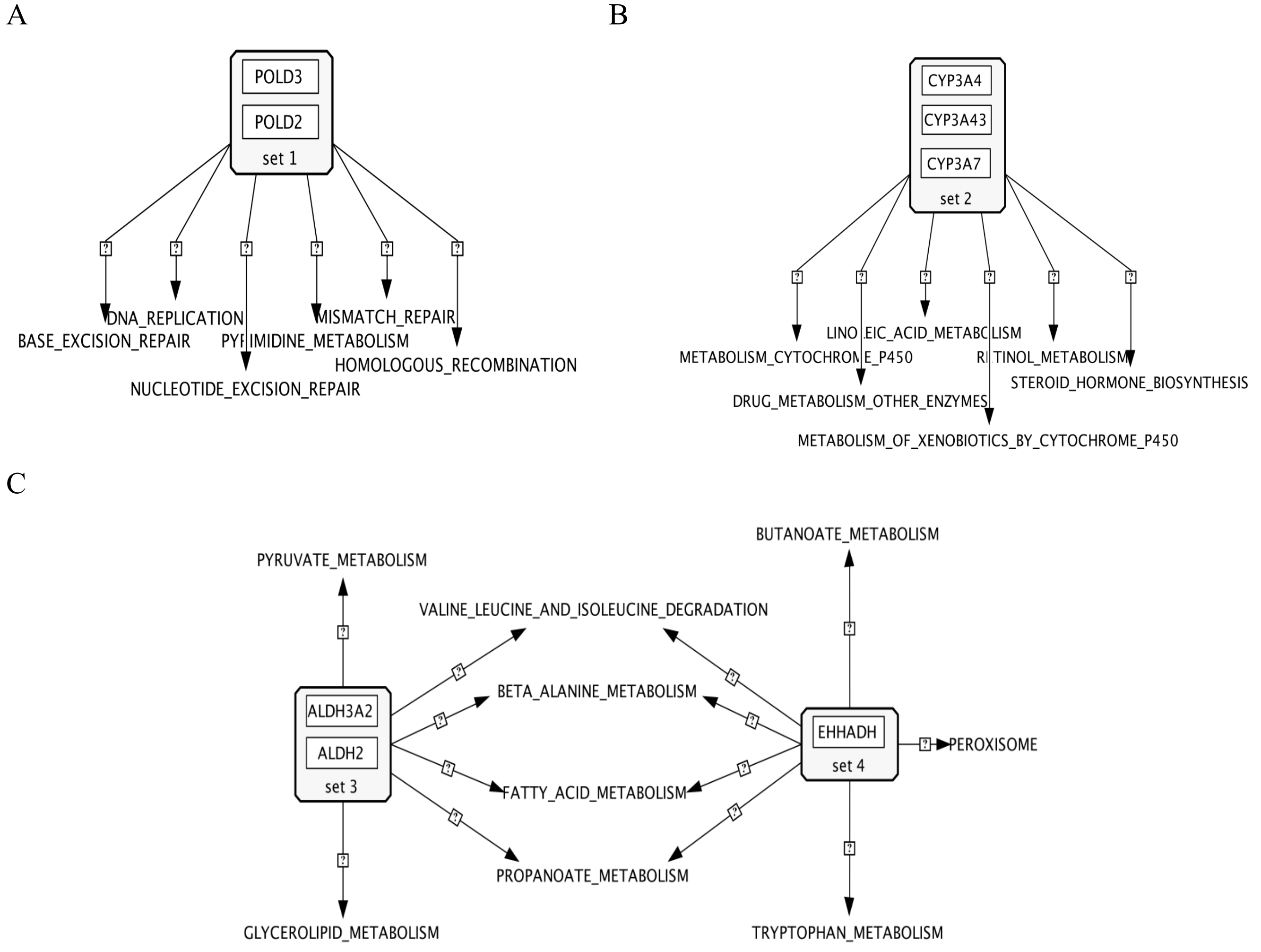


Figure 1 The detailed top common genes of POLD (A), CYP3A (B), ALDH and EHHADH (C) together with their connected pathways in the biochips group using gene expression data.
