# Supplementary material for: Zonation related function and ubiquitination regulation in human hepatocellular carcinoma cells in dynamic vs. static culture conditions
Source: BMC Genomics. 2012 Feb 1;13:54. doi: 10.1186/1471-2164-13-54 (PMC3295679; doi:10.1186/1471-2164-13-54)
Supplement: Additional file 4 — Figure S2: Painted WNT signaling pathway based on the Petri gene expression data using Paintomics. Genes with values higher than the mean value of all gene expressions are colored in red and otherwise in blue, while the intensity is proportional to the differentially expressed level. The image shows the link-outs to other pathways as well. [file 1471-2164-13-54-S4.PDF]

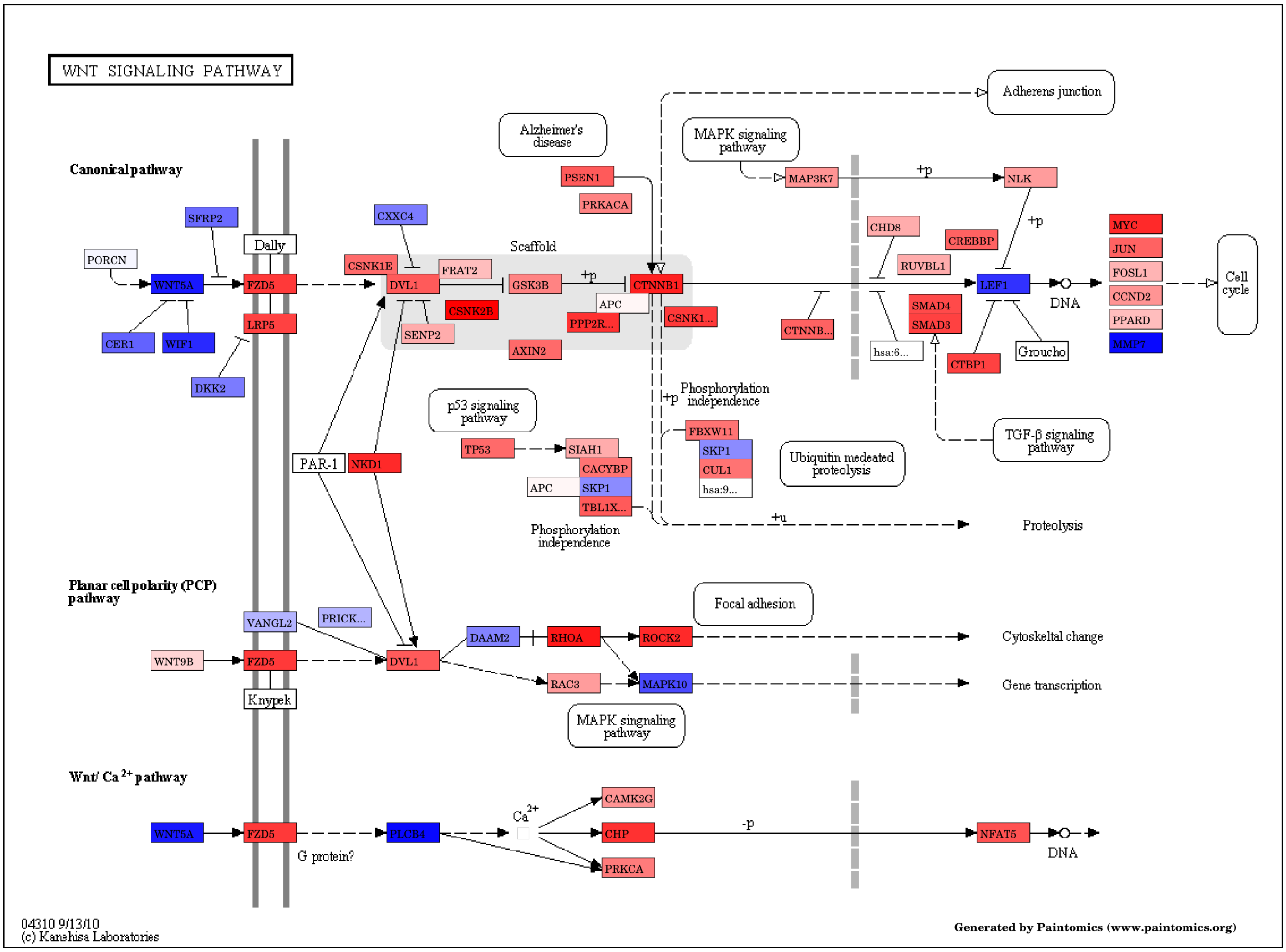

Figure 2 Painted WNT signaling pathway based on the Petri gene expression data using Paintomics. Genes with the values more than the mean value of all gene expression are colored in red and otherwise in blue, while the intensity is proportional to the differentially expressed level. The image shows the link-outs to other pathways as well.
