# Supplementary material for: Zonation related function and ubiquitination regulation in human hepatocellular carcinoma cells in dynamic vs. static culture conditions
Source: BMC Genomics. 2012 Feb 1;13:54. doi: 10.1186/1471-2164-13-54 (PMC3295679; doi:10.1186/1471-2164-13-54)
Supplement: Additional file 5 — Table S3: Proteins differentially expressed by the biochips versus the Petri via 2D DIGE technique. [file 1471-2164-13-54-S5.PDF]

Table 3 Proteins differentially expressed by the biochips versus the Petri via 2D DIGE technique.

Identifiant swissprot	Gene Name	Complete Name	Accession number swiss prot	Fold change
ACDSB_HUMAN	ACADSB	Short/branched chain specific acyl-CoA dehydrogenase, mitochondrial	P45954	-1,8
THIC_HUMAN	ACAT1	Acetyl-CoA acetyltransferase	Q9BWD1	1,5
ATCG_HUMAN	ACTG1	Actine, cytoplasmique	P63261	1,7
FETA_HUMAN	AFP	Alpha-fetoprotein	P02771	3,4
ALBU_HUMAN	ALB	Serum albumin	P02768	2,3
AL1A1_HUMAN	ALDH1A1	Retinal dehydrogenase 1	P00352	2
AL1B1_HUMAN	ALDH1B1	Aldehyde dehydrogenase X mitochondrial	P30837	1,4
AMGO2_HUMAN	AMIGO2	Amphoterin-induced protein 2	Q86SJ2	-1,8
ANXA6_HUMAN	ANXA6	Anexin A6	P08133	1,5
ANXA7_HUMAN	ANXA7	Annexin A7	P20073	-2,6
KCRB_HUMAN	CKB	Creatine kinase B	P12277	-2,7
COR1B_HUMAN	CORO1B	Coronin-1B	Q9BR76	-1,8
CATB_HUMAN	CTSB	Cathepsine B	P07858	2
CATB_HUMAN	CTSB	Cathepsine B	P07858	-2,4*
CATC_HUMAN	CTSC	Dipeptidyl-peptidase 1	P53664	-1,4
CATD_HUMAN	CTSD	Cathepsine D	P07339	-2,1
P07339	CTSD	Cathepsin D	P07339	-1,6
SRC8_HUMAN	CTTN	Src substrate cortactin	Q14247	-1,7
DD19B_HUMAN	DDX19B	ATP-dependent RNA helicaseDDX19B	Q9UMR2	2,7
ODP2_HUMAN	DLAT	Dihydrolipoyllysine-residue acetyltransferase component of pyruvate dehydrogenase complex	P10515	1,8
DPP4_HUMAN	DPP4	Dipeptidyl peptidase 4	P27487	-1,9
E2F8_HUMAN	E2F8	Transcription factor E2F8	A0AVK6	1,9
EF2_HUMAN	EEF2	Elongation Factor 2	P13639	1,5*
EIF3F_HUMAN	EIF3F	Eukaryotic translation initiation factor 3 subunit F	O00303	-2,1
EiF5A1	EIF5A	Eukaryotic translation initiation factor 5A-1	P63241	1,5
ENOA_HUMAN	ENO1	Alpha-enolase	P06733	-1,7
ENOG_HUMAN	ENO2	Gamma-enolase	P09104	-2,1
ERO1A_HUMAN	ERO1L	ERO1-like protein alpha	Q96HE7	-2,1
ERP29_HUMAN	ERP29	Endoplasmic reticulum resident protein 29	P30040	1,6
FRIL_HUMAN	FTL	(Ferritin light chain)	P02792	1,7
	GALM	Aldose 1 epimérase	Q96C23	1,5
GRPL2_HUMAN	GLIPR1L2	GLIPR1-like protein2	Q4G1C9	-1,6
GLSK_HUMAN	GLS	Glutaminase kidney isoform, mitochondrial	O94925	2,7
GRPE1	GRPEL1	GrpE protein homolog 1	Q9HAV7	1,9
GRSF1_HUMAN	GRSF1	G-rich sequence factor 1	Q12849	1,6
GSTO1	GSTO1	Glutathion transférase omega 1	P78417	2,4
HMCS1_HUMAN	HMGCS1	Hydroxymethylglutaryl-CoA synthase, cytoplasmic	Q01581	2,5
HMCS1_HUMAN	HMGCS1	Hydroxymethylglutaryl-CoA synthetase	Q01581	-1,4
HNRPC_HUMAN	HNRPC	Heterogeneous nuclear ribonucleoproteins C1/C2	P07910	1,9*
HS90A_HUMAN	HSP90AA1	Heat shock protein HSP 90-alpha	P07900	1,6
HS90B_HUMAN	HSP90AB1	Heat shock protein HSP 90-beta	P08238	1,6
ENPL_HUMAN	HSP90B1	Endoplasmin	P14625	-1,8
GRP78_HUMAN	HSPA5	78 kDa glucose-regulated protein	P11021	-1,7
HSP7C_HUMAN	HSPA8	Heat shock cognate 71 kDa protein	P11142	1,6
IDE_HUMAN	IDE	Insulin-degrading enzyme	P14735	1,8
K2C1_HUMAN	KRT1	Keratin, type II cytoskeletal 1	P04264	1,5
K2C1_HUMAN	KRT1	Keratin, type II cytoskeletal 1	P04264	-1,8
K1C18_HUMAN	KRT18	Keratin, type I cytoskeletal 18	P05783	-1,6
K2C8_HUMAN	KRT8	Keratin, type II cytoskeletal 8	P05787	-2,1*
LACB2_HUMAN	LACTB2	Beta-lactamase-like protein 2	Q53H82	-1,8
LEG12_HUMAN	LGALS12	Galectin-12	Q96DT0	-1,6

MCM7_HUMAN	MCM7	DNA replication licensing factor MCM7	P33993	1,6
RM12_HUMAN	MRPL12	39S ribosomal protein L12	P52815	1,8
NACA_HUMAN	NACA	Nascent polypeptide-associated complex subunit alpha	Q13765	2,5
NUCL_HUMAN	NCL	Nucleolin	P19338	1,7
NDRG1_HUMAN	NDRG1	Protein NDRG1	Q92597	-4,3
NDUS1_HUMAN	NDUFS1	NADH-ubiquinone oxidoreductase 75 kDa subunit	P28331	-1,6
NT5C_HUMAN	NT5C	5'(3')-deoxyribonucleotidase, cytosolic type	Q8TCD5	-2,1
PAIP1_HUMAN	PAIP1	Polyadenylate-binding protein-interacting protein 1	Q9H074	1,6
PARK7	PARK7	Protéine DJ-1	Q99497	2
PCNA_HUMAN	PCNA	Proliferating cell nuclear antigen	P12004	2,3
PDIA3_HUMAN	PDIA3	Protein disulfide-isomerase A3	P30101	-1,5
PHB	PHB	Prohibitin	P352 32	2,4
SERA_HUMAN	PHGDH	D-3-phosphoglycerate dehydrogenase	O43175	2,1
M6PBP_HUMAN	PLIN3	Mannose-6-phosphate receptor-binding protein 1	O60664	-2,1
POTEE_HUMAN	POTEE	POTE ankyrin domain family member E	Q6S8J3	1,7
RBBP4_HUMAN	RBBP4	Histone-binding protein RBBP4	Q09028	1,6
RCN2_HUMAN	RCN2	Reticulocalbin-2	Q14257	2
RING2_HUMAN	RNF2	E3 ubiquitin-protein ligase RING2	Q99496	-1,5
BRE1A_HUMAN	RNF20	E3 ubiquitin-protein ligase BRE1A	Q5VTR2	-1,6
S100P_HUMAN	S100P	Protein S100-P	P25815	-2,2
DHSA_HUMAN	SDHA	Succinate dehydrogenase [ubiquinone] flavoprotein subunit	P31040	-2
SEP14_HUMAN	SEPT14	Septin-14	Q6ZU15	-1,5
SPB8_HUMAN	Serpin B8	Serpin B8	P50452	-1,9
A1AT_HUMAN	SERPINA1	Alpha-1-antitrypsin	P0109	3,5
ILEU_HUMAN	SERPINB1	Leukocyte elastase inhibitor	P30740	-1,9
1433S_HUMAN	SFN	14-3-3 protein sigma	P31947	1,5
SPRC_HUMAN	SPARC	SPARC	P09486	1,5*
SPRC_HUMAN	SPARC	SPARC	P09486	-2,1*
SPTA2_HUMAN	SPTAN1	Spectrin alpha chain, Brain	Q13813	-1,8
ST1A1_HUMAN	ST1A1	Sulfotransférase 1A1	P50225	1,5
STRAP_HUMAN	STRAP	Serine-threonine kinase receptor-associated protein	Q9Y3F4	1,4
TALDO_HUMAN	TALDO1	Transaldolase	P37837	1,5
TIM50_HUMAN	TIMM50	Mitochondrial import inner membrane translocase subunit TIM50	Q3ZCQ8	1,6
TPIS_HUMAN	TPI1	Triosephosphate isomerase	P60174	-1,9
TBA1B_HUMAN	TUBA1B	Tubulin alpha-1B chain	P68363	-1,6
TRXR1_HUMAN	TXNRD1	Thioredoxin reductase 1, cytoplasmic	Q16881	1,9/3,1
VAT1_HUMAN	VAT1	Synaptic vesicle membrane protein VAT-1 homolog	Q99536	1,6
VISL1_HUMAN	VSNL1	Visinin-like protein 1	P62760	-1,5
1433G_HUMAN	YWHAG	14-3-3 protein gamma	P61981	1,8
